# Oertel’s Microscopy

**Published:** 1903-01

**Authors:** 


Oertel's Microscopy.—Medical microscopy, designed for students in laboratory work and for practitioners. By T. E. Oertel, M. D., Professor of Histology, Pathology, Bacteriology and Clinical Microscopy, Medical Department University of Georgia. With 13 illustrations. P. Blakiston's Sons & Co., Philadelphia, Pa. 1902.

As the author claims, this book is only intended for beginners, or physicians who are compelled to work unaided by an instructor. In this field the volume will be of undoubted value, especially to the rural practitioner who hasn't the advantages of city laboratories. Using it as a guide to laboratory methods he can qualify himself for more thorough training and in the mean time arm himself with certain diagnostic information which he cannot afford to be without if he wishes in any way to approach scientific methods. L.

